# An effective screening tool for uncovering degenerative cervical myelopathy: Results of the prospective multicenter SCREEN-DCM study

**DOI:** 10.1016/j.bas.2026.107478

**Published:** 2026-10-16

**Authors:** Aria Nouri, Nasser Bouhalassa, Simone Grannò, Francesca Brigatti, Granit Molliqaj, Alexandre Lavé, Gianpaolo Jannelli, Allan Martin, Rebecca Garner, Justin Virojanapa, Joseph Cheng, Teresa Somma, Mark Kotter, Carmen Vleggeert-Lankamp, Karl Schaller, Enrico Tessitore

**Affiliations:** a Division of Neurosurgery, Geneva University Hospitals, Switzerland; b Department of Neurological Surgery, University of California, Davis, Sacramento, CA, USA; c Department of Neurosurgery, University of Cincinnati College of Medicine, Cincinnati, OH, USA; d Division of Neurosurgery, Department of Neurosciences, Reproductive and Odontostomatological Sciences, Università degli Studi di Napoli Federico II, Naples, Italy; e Department of Clinical Neurosciences, University of Cambridge, Cambridge, UK; f Department of Neurosurgery, Leiden University Medical Center (LUMC), Leiden, the Netherlands

**Keywords:** Cervical spondylotic myelopathy, Diagnostic delay, Degenerative cervical myelopathy, Screening, Test

## Abstract

**Background context:**

Degenerative cervical myelopathy (DCM) is a leading cause of spinal cord injury, yet it remains frequently undiagnosed. Earlier identification may help guide patients into appropriate diagnostic and referral pathways before substantial neurological disability has accumulated. This study aimed to evaluate the diagnostic performance of SCREEN-DCM for identifying previously undiagnosed DCM.

**Material and methods:**

We conducted a prospective multicenter diagnostic accuracy study, where 200 consecutive adults presenting with lumbar degenerative disease were screened with the SCREEN-DCM clinical score, based on established DCM symptoms, signs, and risk factors. SCREEN-DCM scores ≥3 were considered positive.

**Results:**

69 (34.5%) participants screened positive and 131 (65.5%) screened negative. Cervical MRI was performed in 83 participants. DCM was identified in 29 of 57 screen-positive patients and 1 of 26 screen-negative patients. SCREEN-DCM demonstrated 96.7% sensitivity, 47.2% specificity, 50.9% positive predictive value, 96.2% negative predictive value, and an AUC of 0.87 (95% CI, 0.79-0.95). Approximately two MRIs were required to detect one DCM case among screen-positive patients. Diagnostic sensitivity remained high across exploratory age groups. A simplified eight-variable model showed comparable discrimination (AUC, 0.85; 95% CI, 0.77-0.93), with Hoffman's sign, gait ataxia, and hand clumsiness among the strongest predictors of DCM.

**Conclusions:**

SCREEN-DCM has a very high sensitivity to identify DCM in previously undiagnosed patients. By identifying patients who may otherwise experience diagnostic delay, the screening tool could support earlier referral and prevention of avoidable neurological disability.

## Highlights

• SCREEN-DCM detected previously undiagnosed degenerative cervical myelopathy.
• Almost 1 in 2 positive patients had degenerative cervical myelopathy.
• High sensitivity made the tool suitable for screening.
• Screening may shorten diagnostic delay and support earlier specialist referral.

## Graphical abstract

**Figure:**
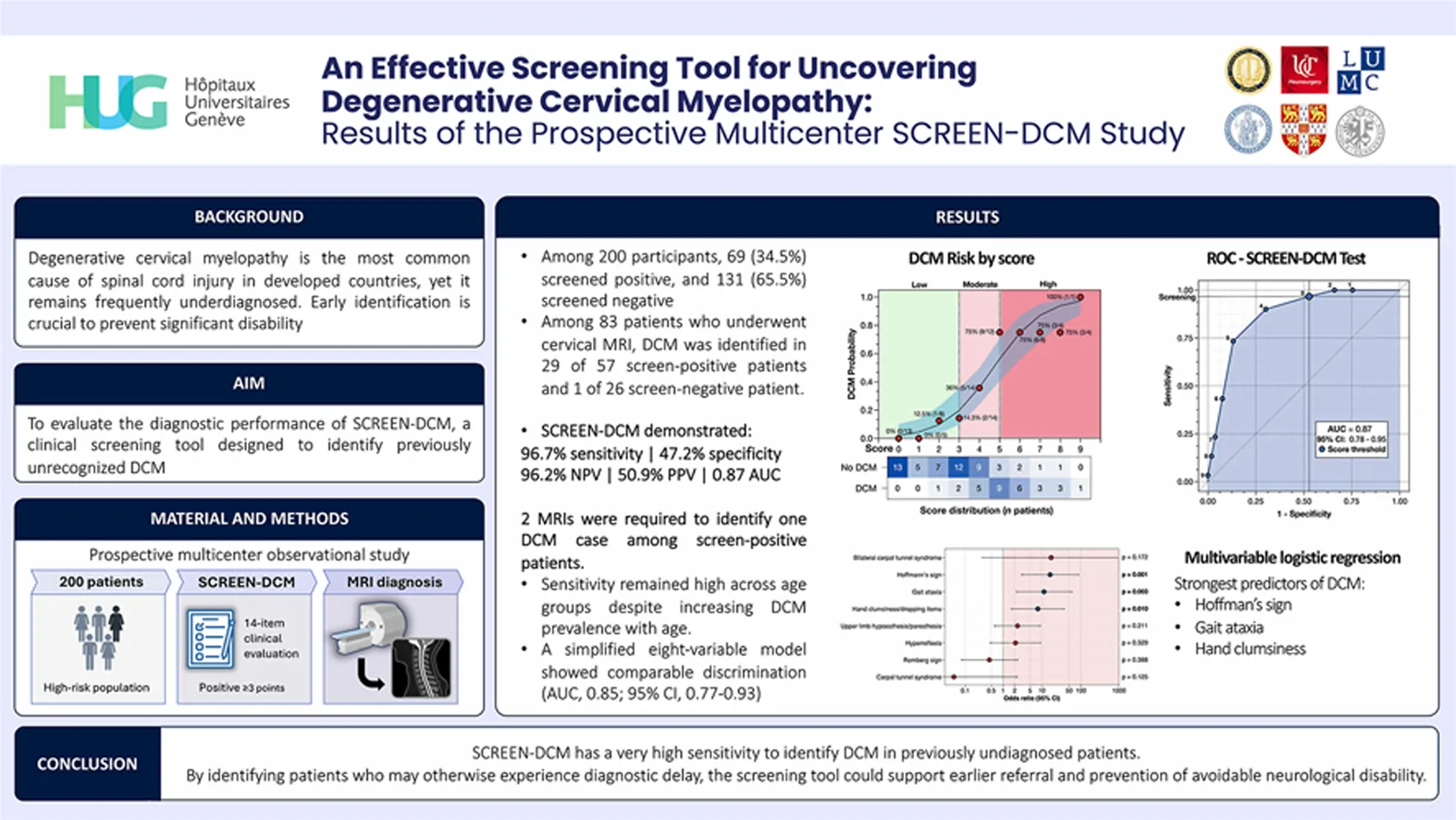

## 1 Introduction

Degenerative Cervical Myelopathy (DCM) is a progressive and frequently under-recognized cause of adult spinal cord dysfunction (Nouri et al., 2015, 2020). Although it arises from degenerative compression of the cervical spine, its clinical presentation is often broad and nonspecific. Patients may report hand clumsiness, gait imbalance, falls, sensory disturbance, or loss of functional independence, symptoms that can easily be misattributed to aging or other musculoskeletal conditions (Gibson et al., 2018).

Chronic progression, confounding comorbidities, and limited recognition outside specialist settings routinely delay diagnosis. This delay directly increases disease severity at the time of specialist consultation (Hilton et al., 2019; Behrbalk et al., 2013). In older adults, undiagnosed DCM may contribute to falls and central cord syndrome; nearly 20% of patients with hip fractures were identified to have undiagnosed DCM in one study (Singh et al., 2014; Radcliff et al., 2016). From a health-system perspective, these missed or delayed diagnoses represent an opportunity for earlier detection during routine encounters.

Taken together, these challenges support a clear role for structured clinical screening to improve early recognition of DCM. Screening tools are most effective when applied to high-risk populations, such as patients that are already followed in clinic for degenerative disease of the lumbar spine. Indeed, the prevalence of tandem stenosis (both lumbar and cervical stenosis) has been estimated between 5 and 28% (LaBan and Green, 2004; Jannelli et al., 2019). Existing DCM screening approaches have largely focused on questionnaires, with no widely adopted clinical strategy available (Barkoh et al., 2019; Kobayashi et al., 2010). The SCREEN-DCM tool was therefore developed to facilitate earlier DCM recognition by combining established symptoms, signs, and risk factors into a simple clinical score (Nouri et al., 2022a). By focusing on a higher-risk population, the study aims to provide a cost-effective screening strategy and identify the demographic and clinical variables most predictive of DCM, which could direct potential adaptation of the screening tool to larger population screening.

## 2 Material and methods

### 2.1 Study design and participants

The SCREEN-DCM study protocol, rationale, and screening criteria were previously published (Nouri et al., 2022a). This screening tool was developed from established DCM signs, symptoms, and risk factors identified from prior literature (Harrop et al., 2010; Baucher et al., 2022; Nouri et al., 2017; Rhee et al., 2009). This prospective multicenter study recruited consecutive adults presenting with non-cervical degenerative spine disease, primarily lumbar disease, across five sites in Switzerland, Italy, the Netherlands, and the USA between 2021 and 2025. However, due to factors outside of our control, notably BREXIT and COVID, initial plans to include a British site was annulled and the speed of recruitment was impacted.

Exclusion criteria included prior DCM diagnosis or cervical surgery, metastatic spine disease, pregnancy, incarceration, lacked mental capacity or missing screening data. Screening variables and thresholds were established by expert consensus (Nouri et al., 2022a). Each criterion contributed one point (Table 1) and a score ≥3 prompted cervical MRI evaluation. DCM diagnosis was established using combined clinical and radiological assessment by the treating neurosurgeon. Patients with <3 points were not consulted to undertake an MRI; however, we accepted any cervical MRI undertaken after the screening during the study period.

**Table 1:** **Screening criteria for DCM.** *1 point for unilateral carpal tunnel syndrome, 2 points if bilateral. Abbreviation: DCM, degenerative cervical myelopathy.

Signs	Points	Comorbidity	Points
Hyperreflexia	1	Rheumatoid arthritis	1
Hoffman's sign	1	Klippel-Feil syndrome	1
Ankle clonus	1	Carpal tunnel syndrome*	1 or 2
Neck pain	1		
Babinski sign	1		
Upper limb hypoesthesia/paresthesia	1		
Inverted brachioradialis reflex	1		
Gait ataxia	1		
Hand clumsiness/dropping items	1		
Romberg sign	1		

The primary endpoint was to evaluate the performance of the proposed screening test. Secondary endpoints comprised assessing the predictive capacity of individual clinical signs, optimization of the screening-tool and investigation of the impact of age on diagnostic performance.

### 2.2 Descriptive analysis

Patients who received cervical MRIs were included in the diagnostic validation cohort. Continuous variables were presented as means with standard deviations, while categorical variables were reported as counts with percentages. Patient demographics included age, gender, body mass index and smoking status. The distribution of clinical signs and symptoms was compared between patients with and without DCM using the chi-square test or Fisher's exact test.

### 2.3 Diagnostic test performance

The performance of the SCREEN-DCM test was evaluated using a score threshold of ≥3 points for positivity. True positives, false positives, true negatives and false negatives were calculated for the MRI validation cohort. From these, standard diagnostic metrics were computed: sensitivity, specificity, positive predictive value (PPV), negative predictive value (NPV), the Youden's index (J = sensitivity + specificity – 1) and the number needed to diagnose (NND). Univariate diagnostic performance metrics for each individual signs and symptom, were additionally calculated and presented in eTable 2.

Univariate logistic regression assessed the association between SCREEN-DCM score and diagnosis. Odds ratio (OR) and 95% confidence intervals (CI) were calculated, and model calibration was assessed using the Hosmer-Lemeshow test. Discriminatory performance of the test was assessed using ROC analysis with calculation of AUC, sensitivity, specificity, and Youden's index.

Because verification by MRI wasn't available for all patients, diagnostic accuracy was additionally estimated using the Begg-Greenes correction for partial verification, which reweights disease probability within each screening group to the full cohort (Begg and Greenes, 1983). Corrected sensitivity, specificity, prevalence and AUC were reported with 95% CIs from 2000 bootstrap replicates stratified on screening result.

### 2.4 Model optimization

For model optimization, variables with fewer than 5 occurrences in the validation cohort were excluded from further analysis. AIC-guided backward stepwise elimination sequentially removed variables to achieve a parsimonious 8-variable model to minimize overfitting (Sperandei, 2014). Elimination was then continued to assess the contribution of individual variable, recalculating performance metrics at each elimination. The optimized 8-variable model was compared to the full model using the DeLong Test. Multivariate logistic regression was performed on the optimized set of variables to identify independent predictors of DCM. All candidate predictors were binary (defined as present or absent). For each predictor, the OR with 95% CI and p-value were reported.

### 2.5 Assessment of age on screening

To assess age-related variations in diagnostic performance, the MRI cohort was stratified into age groups: <50, 50-59, 60-69, 70-79 and ≥ 80 years. Performance metrics were computed for each stratum using the fixed positivity threshold (score ≥3). The relationship between patient age and SCREEN-DCM score was examined with Spearman rank correlation after rejection of normality by Shapiro-Wilk.

### 2.6 Statistical analysis

All analyses were performed using R (version 4.5.0) with significance determined at p < 0.05.

## 3 Results

### 3.1 Study population and demographics

Of 213 patients enrolled prospectively, 13 were excluded (9 invalid consent, 3 meeting exclusion criteria, 1 loss-to-follow-up), resulting in a final cohort of 200 patients. Based on the predefined cut-off for positivity (score ≥3), 69 patients (34.5%) screened positive and 131 patients (65.5%) screened negative (Fig. 1). Mean age was 61.1 ± 15.4 years, with equal gender distribution. Patients with positive screens were slightly older (62.9 *vs* 60.1 years), had higher BMI (29.9 *vs* 27.9 kg/m^2^), and demonstrated a significantly higher prevalence of hyperreflexia (62.3% vs 6.1%), Hoffman's sign (47.8% vs 1.5%), and gait ataxia (66.7% vs 19.1%) compared to negative-screen patients (Table 2).

**Fig. 1:**
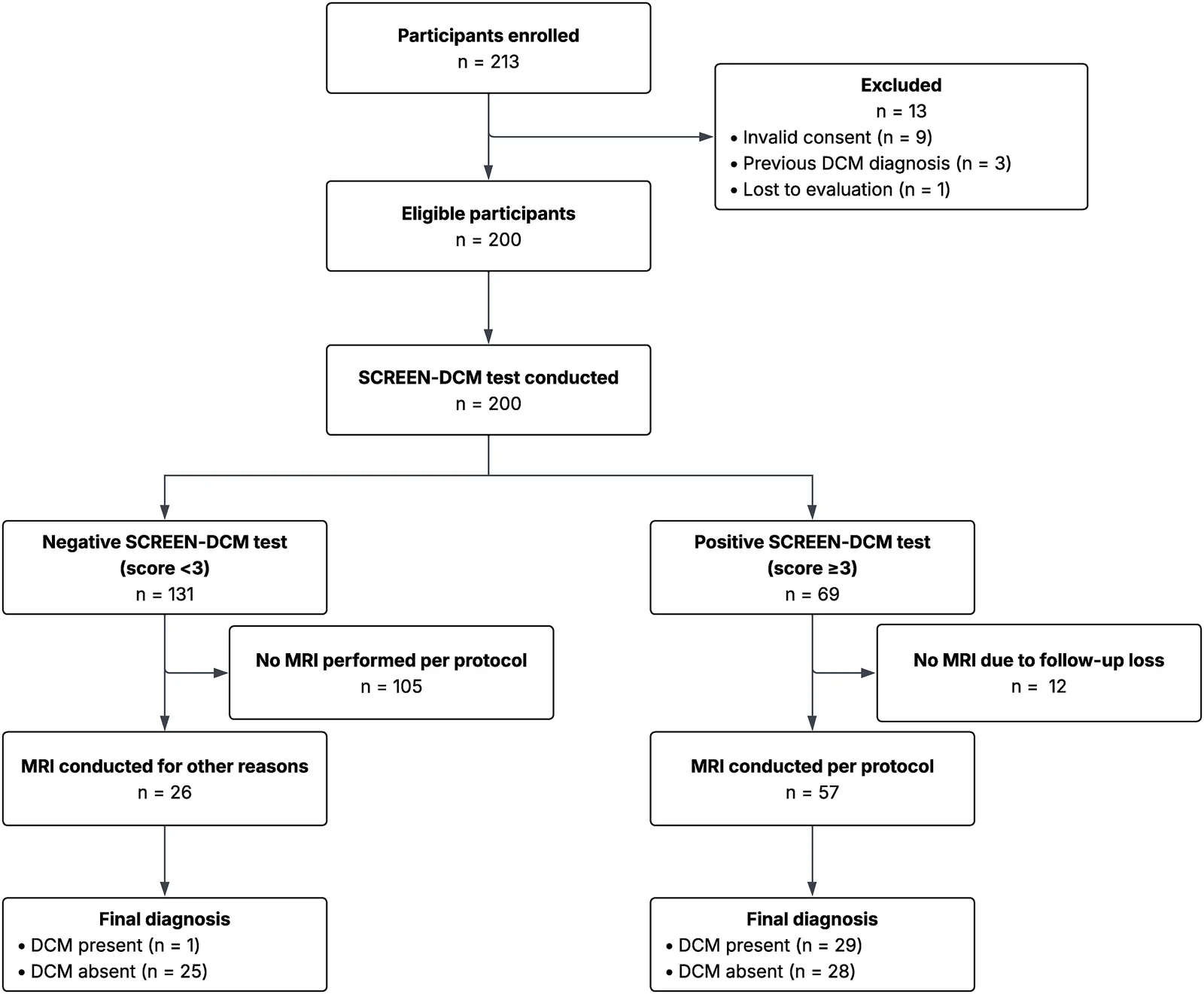
**Flow Diagram of Enrolled Patients**. Abbreviation: DCM, Degenerative cervical myelopathy.

**Table 2:** **Characteristics of enrolled patients.** Abbreviation: SD, standard deviation.

Characteristic	Screening group			p-value
	Score < 3 (n = 131)	Score ≥ 3 (n = 69)	All (n = 200)	
**Age, mean (SD), y**	60.1 (16.5)	62.9 (12.7)	61.1 (15.4)	0.192
**Gender, No. (%)**				0.583
**Female**	63 (48.1)	36 (52.2)	99 (49.5)	
**Male**	68 (51.9)	33 (47.8)	101 (50.5)	
**BMI, mean (SD), kg/m^2^**	27.9 (5.1)	29.9 (7.7)	28.6 (6.2)	0.07
**Smoker, No. (%)**				0.478
**Non smoker**	62 (47.3)	27 (39.1)	89 (44.5)	
**Previous smoker**	26 (19.8)	18 (26.1)	44 (22)	
**Current smoker**	28 (21.4)	14 (20.3)	42 (21)	
**Clinical signs and symptoms, No. (%)**				
**Hyperreflexia**	8 (6.1)	43 (62.3)	51 (25.5)	**<0.001**
**Hoffman's sign**	2 (1.5)	33 (47.8)	35 (17.5)	**<0.001**
**Ankle clonus**	2 (1.5)	7 (10.1)	9 (4.5)	**0.009**
**Neck pain**	23 (17.6)	49 (71)	72 (36)	**<0.001**
**Babinski sign**	0 (0)	6 (8.7)	6 (3)	**0.001**
**Upper limb hypoesthesia/paresthesia**	9 (6.9)	39 (56.5)	48 (24)	**<0.001**
**Inverted brachioradialis reflex**	1 (0.8)	17 (24.6)	18 (9)	**<0.001**
**Gait ataxia**	25 (19.1)	46 (66.7)	71 (35.5)	**<0.001**
**Hand clumsiness (dropping items)**	0 (0)	30 (43.5)	30 (15)	**<0.001**
**Romberg sign**	8 (6.1)	23 (33.3)	31 (15.5)	**<0.001**
**Rheumatoid arthritis**	3 (2.3)	1 (1.4)	4 (2)	1
**Klippel-Feil syndrome**	0 (0)	2 (2.9)	2 (1)	0.119
**Carpal tunnel syndrome**	5 (3.8)	15 (21.7)	20 (10)	**<0.001**
**Bilateral carpal tunnel syndrome**	1 (0.8)	10 (14.5)	11 (5.5)	**<0.001**

The diagnostic validation cohort comprised 83 patients who underwent cervical MRI: 57 of 69 screen-positive patients (82.6%) per protocol, and 26 screen-negative patients imaged for independent clinical indications (Fig. 1). Patients diagnosed with DCM were older (68.6 vs 60.9 years) and had higher screening scores (5.4 vs 2.5) than non-DCM patients (eTable 1). Hoffman's sign, gait ataxia, hyperreflexia, hand clumsiness, upper limb sensory changes, and inverted brachioradialis reflex were significantly more prevalent in the DCM group (eFig. 1, eTable 2).

### 3.2 Test performance and diagnostic accuracy

The screening test demonstrated excellent sensitivity with more modest specificity, 96.7% (88.9-100%) and 47.2% (35.3-58.8%) respectively. The PPV was 50.9% (37.7-64.4%) and the NPV was 96.2% (87.5-100%), indicating that a negative screen was highly effective at excluding DCM, with only 1 false negative among 26 patients with a score under 3. The NND was 2.28.

Analysis of the relationship between SCREEN-DCM score and DCM probability demonstrated a clear, non-linear increase in risk (Fig. 2A). Observed probability rose from 0% at scores 0-1 to 14.3% (score 3), 35.7% (score 4), and ≥75% (scores ≥5). Logistic regression confirmed a strong association with a 2.29-fold increase in odds of DCM (OR 2.29; 95% CI: 1.58-3.32, p < 0.001). Scores ≤3 were associated with low probability of DCM (∼7.5%), score = 4 with intermediate probability (∼35.7%), and scores ≥5 with high probability of DCM (∼75.9%). The model demonstrated adequate calibration (Hosmer-Lemeshow p = 0.407), supporting the ability of the SCREEN-DCM score to stratify patients according to DCM risk. The AUC for the SCREEN-DCM score was 0.87 (95% CI: 78.7-93.5%, Fig. 2B), indicating excellent discriminatory performance.

**Fig. 2:**
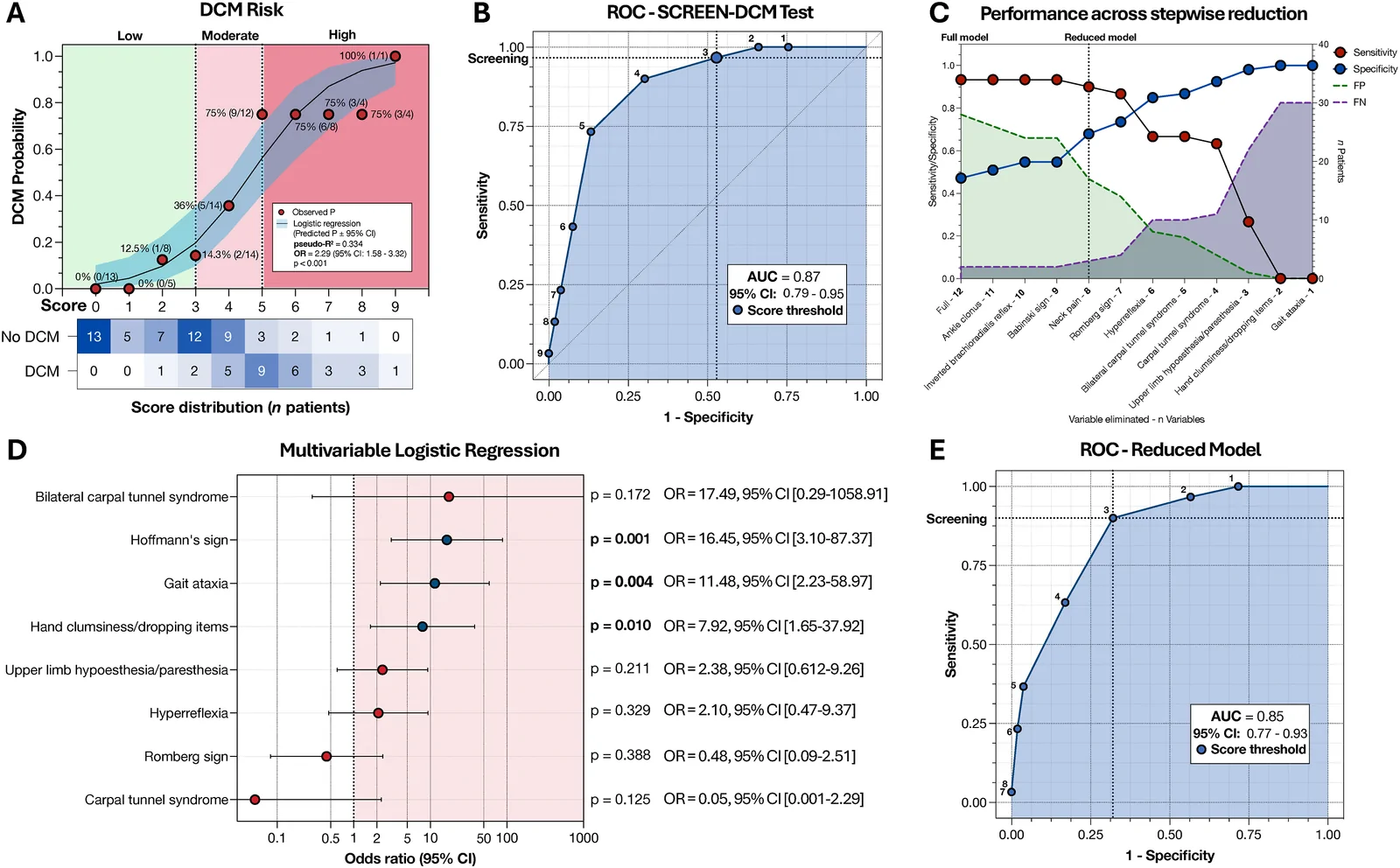
**Performance of the SCREEN-DCM Test.** Probability of DCM according to SCREEN-DCM score, modelled using logistic regression (A). ROC curve demonstrating the discriminatory performance of the SCREEN-DCM test (B). Performance metrics across stepwise model reduction (C). Multivariable logistic regression identifying independent clinical predictors of DCM (D). ROC curve of the reduced 8-variable model demonstrating comparable performance with fewer variables (E).

### 3.3 Model optimization

A more parsimonious model was evaluated through variable elimination starting from the 14 clinical variables; however, variables with n < 5 in the MRI validation cohort were excluded. The AIC-based elimination process then systematically removed variables with minimal predictive contribution, the elimination process converged at eight variables.

Model stability was assessed through stepwise variable elimination (Fig. 2C). Model performance remained stable following removal of several variables, with sensitivity largely unchanged until nine variables remained, while specificity increased progressively in parallel. However, the eight-variable model marked a reversal in sensitivity and specificity. Although false negatives remained minimal up to six-eight variables, further removal below six variables resulted in declining sensitivity and increased missed diagnosis.

The eight-variable model consisted of: hyperreflexia, Hoffman's sign, upper limb hypoesthesia/paresthesia, gait ataxia, hand clumsiness (dropping items), Romberg sign, carpal tunnel syndrome and bilateral carpal tunnel syndrome. Among the eight retained variables, multivariate logistic regression identified Hoffman's sign (OR 16.45, 95% CI: 3.10-87.37, p = 0.001), gait ataxia (OR 11.48, 95% CI: 2.23-58.97, p = 0.004) and hand clumsiness (OR 7.92, 95% CI: 1.65-37.92, p = 0.010). Other variables were not independently associated with DCM (Fig. 2D).

The eight-variable model achieved a sensitivity of 90.0%, specificity of 67.9% PPV of 61.4%, NPV of 92.3%, Youden's index of 0.579 and AUC of 0.85 (95% CI: 0.77-0.93) (Fig. 2E). The DeLong test comparing the ROC curves of the full 14-variables model and the 8-variable model yielded a Z-statistic of 0.9597 (p = 0.337), indicating no statistically significant difference in discriminatory performance between the models. The reduce 8-variable model thus achieves comparable diagnostic performance with greater clinical simplicity and reduced assessment burden.

### 3.4 Correction for partial verification

Cervical MRI was obtained in 82.6% of screen-positive but only 19.8% of screen-negative patients, raising the possibility of partial verification bias. Applying the Begg-Greenes correction, which reweights disease probability within each screening group to the full cohort (n = 200), the estimated DCM prevalence was 20.1% (95% CI: 13.9-27.0) rather than the 36.1% observed in the imaged subgroup. Corrected sensitivity was 87.4% (95% CI: 67.1-100) and corrected specificity was 78.8% (95% CI: 74.5-83.4). PPV and NPV remained unchanged as these are directly estimable within screening groups. The corrected AUC was 0.93 (95% CI: 0.88-0.98).

### 3.5 Impact of age on test performance

Analysis of the entire cohort (n = 200) revealed a weak but significant positive correlation between age and SCREEN-DCM score (Spearman ρ = 0.19, 95% CI: 0.061-0.322, p = 0.006, Fig. 3A). Among patients with score ≥3, the correlation was stronger (Spearman ρ = 0.29, 95% CI: 0.07-0.49, p = 0.014, Fig. 3B), suggesting a stronger relationship between age and clinical score within a higher-risk subgroup.

**Fig. 3:**
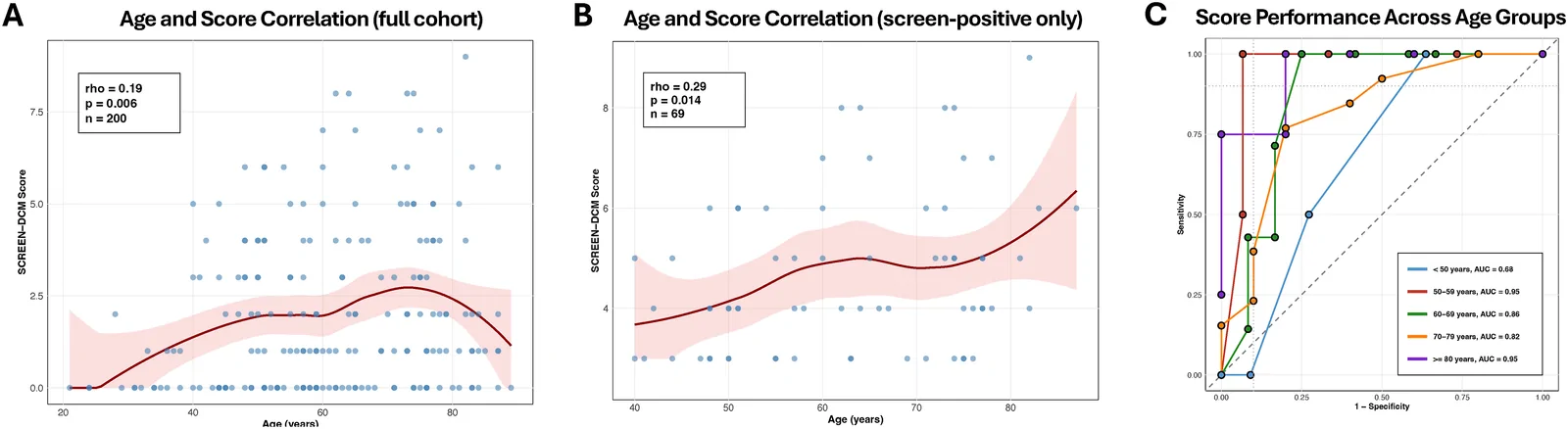
**Impact of age on SCREEN-DCM scores and test performance.** Scatter plot showing the relationship between age and SCREEN-DCM score in the full cohort (n = 200), red line represents a LOESS (locally estimated scatterplot smoothing) curve, with the shaded area indicating the 95% confidence interval (A). Scatter plot of age versus SCREEN-DCM score among screen-positive participants only (n = 69), red line is a LOESS-smoothed curve (B). ROC curve demonstrating the discriminatory performance of the SCREEN-DCM test in each age groups (C).

To assess whether diagnostic performance varied across ages, the MRI validation cohort was stratified into five age groups (Fig. 3C). The DCM prevalence increased with age: 15.4% in patients <50 years, 21.1% in those 50-59 years, 36.8% in those 60-69 years, 56.5% in those 70-79 years, and 44.4% in those ≥80 years. Sensitivity remained consistently high across all age groups (92-100%). Specificity was lowest in younger age groups (36% in <50 years, 33% in 50-59 years) and improved with advancing age (58% in 60-69 years, 50% in 70-79 years, 80% in ≥80 years). The discriminatory performance, as reflected by AUC, was lowest in the youngest group (0.68 in <50 years) and highest in the 50-59 years and ≥80 years groups (both 0.95). AUC ranged from 0.81 to 0.86 in patients age 60-79 years (Fig. 3C). These findings suggest that the SCREEN-DCM test performs well across age groups.

## 4 Discussion

Despite being a significant burden to society, DCM remains frequently under-recognized, particularly when patients present with nonspecific neurological or musculoskeletal complaints. In this prospective multicenter study, the developed screening tool has demonstrated that DCM can be effectively uncovered in patients with high confidence as evidenced by the high sensitivity and negative predictive value of the test. Further, it has been demonstrated that the sensitivity remains unaffected by age despite the increase of prevalence of DCM amongst elderly patients, showing that the screening tool provides consistent detection performance regardless of age. Similarly important is the ability of a screening test to be efficient from both a clinical standpoint and economic standpoint, and the results indicate that it required about 2 screening tests for 1 confirmed diagnosis of DCM (NND = 2.28), suggesting an efficient use of resources.

The effectiveness of screening tools is placed primarily on their ability to discover those with disease, whereas higher specificity is sought for confirming diseases (Aggarwal et al., 2022). We demonstrated that the hypothesized cut-off of 3 or more points, which was initially based on limited research and clinical judgment was indeed an optimal cut-off point. Reducing the threshold to 2 points would have increased sensitivity at the expense of specificity and MRI efficiency.

### 4.1 Screening as a strategy to reduce diagnostic delay

DCM imposes a significant burden of disability and reduced quality of life, estimated only to be superseded by congestive heart failure (Oh et al., 2017). Diagnostic delays have been shown to result in patient presenting with worse initial clinical severity and given that this relates directly to prognosis, this results in worse clinical outcomes (Pope et al., 2020). A key contributor to these delays and to the misdiagnoses, is the lack of clear diagnostic criteria for DCM, hindering timely referral, a challenge highlighted in recent work from the AOSpine (Hilton et al., 2022). Consequently, such delays may result in irreversible disability leading to premature mortality, a risk that could be mitigated with earlier intervention (Davies et al., 2023). Screening provides an opportunity to address these challenges, as the primary objective is identify previously unrecognized disease early in patients who are not yet within a DCM diagnostic pathway. Indeed, none of the patients in the current study were addressed for DCM, and therefore, it is safe to assume that their recognition would have at best been delayed substantially. It is likewise safe to assume that the majority of these patients did not suffer from severe DCM as this represents a severe disability and would likely have been investigated specifically prior, indicating that the vast majority of uncovered diagnosis were milder. Indeed, while it was not a primary objective of this study, it was observed from the confirmed DCM population by the primary investigator that the majority suffered from mild DCM. This finding requires further in-depth exploration as identification of the mild population is key to improving outcomes and evaluating the natural history for those individuals that proceed with non-surgical treatment. These points remain key areas which require optimization in DCM management.

### 4.2 Optimization of screening criteria

Despite the confirmation that the initial cut-off of three or more points provided an excellent and optimal point of differentiation (AUC = 0.87), it is clear that no screening tool can be perfect. Balance must be provided between the use of resources, variations in clinical presentations, adoption from clinicians, ease of use, and economic benefits. When assessing alternative cut-offs important impacts were noted. At a two-point cut-off, the single patient with a false negative in this study was captured, but this resulted in significant inefficiencies to the screening test that may make such criteria inefficient. Likewise, a cut-off of four or five points, results in a higher overall test accuracy, but the resulting substantial gain in specificity came at an important loss in sensitivity, reducing the primary objective of any screening tool.

As a consequence, one must consider that a cut-off of three points may be a strong general point, but the screening criteria at which clinicians may choose to opt for a cervical MRI may vary based on resource availability. Importantly, the clinical utility of a screening test is not determined solely by its intrinsic characteristics, but also the underlying prevalence of disease in the population being tested, which directly impacts predictive value and overall efficiency. In this study, the application in a high-risk population likely contributes to the favorable performance observed (Maxim et al., 2014). Separately, from a practical standpoint, a higher cut-off criterion such as four or five may be more adapted in countries with limited resources, whereas the significant costs of disability associated with DCM in high income countries may sway the economic benefit towards a lower cut-off point. Simplified versions of the tool may improve feasibility while maintaining diagnostic performance, although external validation remains necessary.

### 4.3 Applicability across age groups

While the average age of patients with DCM is in the 60s (Nouri et al., 2022b), this study was designed to screen patients from 18 years of age with the purpose of assessing whether screening criteria would be applicable independent of age. Despite a modest association between age and screening score, diagnostic performance remained stable across age groups. This is important for implementation because DCM may be overlook both in older patients, in whom symptoms may be attributed to aging, and in younger patients, in whom the diagnosis may not initially be suspected. However, given the lower prevalence of DCM in younger patients, the efficiency of any screening tool in this population will be limited and will likely result in scores more often under 3 points, and therefore result in additional clinical work, with few diagnoses of DCM uncovered.

### 4.4 Strengths and limitations

This work represents the first prospective and multicenter clinical screening tool to our knowledge to effectively uncover DCM providing potential applicability across different demographics and backgrounds. A key strength is that this study addresses DCM as a diagnostic pathway problem, rather than only as a condition recognized after specialist referral. However, several limitations should be acknowledged.

First, cervical MRI was not systematically performed in all screened patients, introducing potential partial verification bias. After Begg-Greenes correction, sensitivity was lower and specificity substantially higher than the complete-case estimates. Because screen-negative patients were imaged for independent clinical indications and were therefore likely more symptomatic than those not imaged, the corrected sensitivity is best regarded as a conservative lower bound. The corrected estimates rest on a single false-negative case among 26 imaged screen-negative patients and should be considered exploratory.

A second limitation is related to the number of patients screened – DCM is a variable condition, and certain risk factors may have a low prevalence but may be significant indicators for screening purposes in very large cohorts. While we initially aimed to screen 600 patients, which could have provided a higher number of rarer presentations, our study was limited in this regard, and it is suggested that larger studies are undertaken to confirm the presented findings.

## 5 Conclusion

The results of the SCREEN-DCM trial have shown that screening can effectively and efficiently uncover previously unrecognized DCM in patients who were not referred for suspected cervical cord dysfunction. Moreover, screening remained effective across age groups, indicating that pre-test probability did not influence the sensitivity of the test. Its simplicity may also support use by non-specialists, potentially improving early recognition and referral. These findings suggest that structured clinical screening represents a practical and scalable strategy to enhance the early identification of DCM and improve alignment of patients with the appropriate clinical care pathway. Further validation should assess impact on disability progression and resource use.

## Author contribution

Aria Nouri: Conceptualization, Funding acquisition, Investigation, Methodology, Project administration, Writing – original draft, Writing – review & editing. Nasser Bouhalassa: Data curation, Formal analysis, Visualization, Writing – original draft, Writing – review & editing. Simone Grannò: Formal analysis, Visualization, Writing – original draft, Writing – review & editing. Francesca Brigatti: Writing – review & editing. Granit Molliqaj: Methodology, Writing – review & editing. Alexandre Lavé: Methodology, Writing – review & editing. Gianpaolo Jannelli: Methodology, Writing – review & editing. Allan Martin: Investigation, Methodology, Writing – review & editing. Rebecca Garner: Writing – review & editing. Justin Virojanapa: Methodology, Writing – review & editing. Joseph Cheng: Investigation, Methodology, Writing – review & editing. Teresa Somma: Investigation, Methodology, Writing – review & editing. Mark Kotter: Methodology, Writing – review & editing. Carmen Vleggeert-Lankamp: Investigation, Methodology, Writing – review & editing. Karl Schaller: Methodology, Writing – review & editing. Enrico Tessitore: Methodology, Supervision, Writing – review & editing.

## Ethics declaration

Written informed consent to take part in the study and to publish the article has been obtained from all participants or their legal representatives. The privacy rights of participants have been observed.

This study was performed in compliance with relevant laws, regulatory frameworks and guidelines where the research took place. This study was conducted in accordance with the Standards for the Reporting of Diagnos Accuracy Studies (STARD) guidelines. This study was approved by the Commission cantonale d'éthique de la recherche (CCER). (Approval No. BASEC-2020-02785)

## Disclosure

Enrico Tessitore reports consulting fees from Brainlab, Medability, and Spine Vision; training fees from Depuy Synthes, Medtronic, and Spineart; faculty roles with the EANS Spine Section, Medical AI, AO Spine, EANS, and Eurospine. All other authors declare no competing interests.

## Ethics approval

The study was approved by ethics review (BASEC-2020-02785).

## Funding

The study was supported from funds by CSRS-Europe and EANS Research Awards. A congress abstract presentation was financially supported by the 10.13039/501100015074Swiss Study Foundation.

## Declaration of competing interest

The authors declare that they have no known competing financial interests or personal relationships that could have appeared to influence the work reported in this paper.
