# Supplementary material for: An effective screening tool for uncovering degenerative cervical myelopathy: Results of the prospective multicenter SCREEN-DCM study
Source: Brain Spine. 2026 Oct 16;6:107478. doi: 10.1016/j.bas.2026.107478 (PMC13744266; doi:10.1016/j.bas.2026.107478)
Supplement: Supplementary file 1 [file mmc1.docx]

| **Characteristic** | **MRI diagnosis group** | |
| --- | --- | --- |
|  | **DCM**  **(n = 30)** | **No DCM**  **(n = 53)** |
| **Age, mean (SD), y** | 68.6 (11) | 60.9 (12.9) |
| **Sex, No. (%)** |  |  |
| **Female** | 15 (50) | 21 (39.6) |
| **Male** | 15 (50) | 32 (60.4) |
| **BMI, mean (SD), kg/m^2^** | 29.2 (5.9) | 29.7 (8) |
| **Smoker, No. (%)** |  |  |
| **Non smoker** | 7 (23.3) | 19 (35.8) |
| **Previous smoker** | 11 (36.7) | 8 (15.1) |
| **Current smoker** | 4 (13.3) | 14 (26.4) |
| **SCREEN-DCM score, mean (SD)** | 5.4 (1.6) | 2.5 (2) |

**eTable 1. Characteristics of the MRI validation cohort.**

Abbreviation: SD, standard deviation.

| **Variable** | **Positive cases, n** | **Sensitivity, %** | **Specificity, %** | **PPV, %** | **NPV, %** |
| --- | --- | --- | --- | --- | --- |
| **Gait ataxia** | 46 | 83.3 | 60.4 | 54.3 | 86.5 |
| **Hyperreflexia** | 40 | 73.3 | 66 | 55 | 81.4 |
| **Hoffman’s Sign** | 30 | 70 | 83 | 70 | 83 |
| **Neck pain** | 45 | 70 | 54.7 | 46.7 | 76.3 |
| **Upper limb hypoesthesia/paresthesia** | 36 | 66.7 | 69.8 | 55.6 | 78.7 |
| **Hand clumsiness (dropping items)** | 28 | 56.7 | 79.2 | 60.7 | 76.4 |
| **Romberg sign** | 21 | 36.7 | 81.1 | 52.4 | 69.4 |
| **Inverted brachioradialis reflex** | 15 | 33.3 | 90.6 | 66.7 | 70.6 |
| **Carpal tunnel syndrome** | 13 | 16.7 | 84.9 | 38.5 | 64.3 |
| **Bilateral carpal tunnel syndrome** | 8 | 13.3 | 92.5 | 50 | 65.3 |
| **Babinski sign** | 5 | 10 | 96.2 | 60 | 65.4 |
| **Ankle clonus** | 6 | 6.7 | 92.5 | 33.3 | 63.6 |
| **Klippel-Feil syndrome** | 2 | 6.7 | 100 | 100 | 65.4 |
| **Rheumatoid arthritis** | 1 | 0 | 98.1 | 0 | 63.4 |

**eTable 2. Univariate predictive value of each screening items.**

Sensitivity, specificity, PPV, NPV were calculated using 2x2 contingency tables with DCM diagnosis as the reference standard.

Abbreviations: PPV, positive predictive value; NPV, negative predictive value; CI, standard deviation.

**eFigure 1. Barbell plot demonstrating the prevalence of clinical variables by diagnosis.**

Comparison of prevalence of variables between patients with DCM (red) and without DCM (blue). P-values calculated using Chi-square test (or Fisher's exact test when cell count < 5).

Abbreviation: DCM, degenerative cervical myelopathy
